# Acceptor defects in polycrystalline Ge layers evaluated using linear regression analysis

**DOI:** 10.1038/s41598-022-19221-5

**Published:** 2022-09-02

**Authors:** Toshifumi Imajo, Takamitsu Ishiyama, Koki Nozawa, Takashi Suemasu, Kaoru Toko

**Affiliations:** 1 Institute of Applied Physics, University of Tsukuba, 1-1-1 Tennodai, Tsukuba, Ibaraki 305-8573 Japan; 2 JSPS Research Fellow, 8 Ichiban-cho, Chiyoda-ku, Tokyo, 102–8472 Japan

**Keywords:** Electronics, photonics and device physics, Semiconductors

## Abstract

Polycrystalline Ge thin films have recently attracted renewed attention as a material for various electronic and optical devices. However, the difficulty in the Fermi level control of polycrystalline Ge films owing to their high density of defect-induced acceptors has limited their application in the aforementioned devices. Here, we experimentally estimated the origin of acceptor defects by significantly modulating the crystallinity and electrical properties of polycrystalline Ge layers and investigating their correlation. Our proposed linear regression analysis method, which is based on deriving the acceptor levels and their densities from the temperature dependence of the hole concentration, revealed the presence of two different acceptor levels. A systematic analysis of the effects of grain size and post annealing on the hole concentration suggests that deep acceptor levels (53–103 meV) could be attributed to dangling bonds located at grain boundaries, whereas shallow acceptor levels (< 15 meV) could be attributed to vacancies in grains. Thus, this study proposed a machine learning-based simulation method that can be widely applied in the analysis of physical properties, and can provide insights into the understanding and control of acceptor defects in polycrystalline Ge thin films.

## Introduction

Despite Ge being the oldest semiconductor material^1^, its excellent electrical and optical properties make it a promising next-generation material for various electronic devices, such as transistors^2–4^, solar cells^5,6^, optical communications^7–9^, and thermoelectric devices^10,11^. Multi-junction solar cells are a good example of the practical use of Ge; however, the cost of single-crystal Ge (sc-Ge) substrates limits their application to space use only. On the other hand, Ge is inherently more suitable for applications in thin films than in bulk for the following reasons: (i) Ge has a high optical absorption coefficient (~ 10^4^ cm^–1^ at 0.8 eV), and therefore, can absorb sufficient amount of light even in a thin film^1^. (ii) The leakage current in transistors owing to the narrow band gap can be solved by thinning the Ge substrate^4,12,13^. (iii) It can be synthesized on large-scale integrated Si circuits as well as other general-purpose substrates, such as glass and plastic, because of its low crystallization temperature and Young’s modulus^14,15^. Therefore, there is a strong demand for high-quality Ge thin-film formation techniques, not only from the perspective of cost reduction, but also device performance. Indeed, sc-Ge is often grown epitaxially in SiO_2_ trenches on Si substrates, which is useful for integrated Ge photodiodes^16–18^. Conversely, Ge films synthesized directly on amorphous insulating substrates become polycrystalline containing various defects, including grain boundaries (GBs)^15^. From numerous theoretical and experimental studies on single-crystal Ge, it is known that defects in Ge, such as vacancies and dangling bonds, act as acceptors^19–27^. This is especially remarkable in polycrystalline Ge (poly-Ge) films, where the hole concentration *p* is typically as high as 10^17^–10^18^ cm^–3^^28–34^. This makes it difficult to control the Fermi level of poly-Ge thin films, which is essential for most semiconductor devices.

We discovered that temperature control during the deposition of amorphous precursors can significantly modulate the grain size in the solid-phase crystallization (SPC) process of Ge films^35^. Ge thin films with large grain sizes have a reduced *p* of 1 × 10^17^ cm^−3^, which is the lowest level for poly-Ge thin films^36^, and also enabled n-type conduction control by impurity doping^37,38^. The carrier mobility reached the highest values (690 and 370 cm^2^ V^–1^ s^–1^ for holes and electrons, respectively) for poly-Ge films, even on a flexible plastic substrate^36,39^. In addition, Sn doping in Ge passivated the acceptor defects and reduced its *p* to the order of 10^16^ cm^–3^^39,40^. However, despite the long history of poly-Ge thin films, the behavior of acceptor defects and their levels has not yet been systematically investigated. One of the reasons for this is that it is difficult to control the quality of poly-Ge thin films significantly. In addition, some techniques to identify acceptor levels are difficult to apply to poly-Ge: some acceptor levels are too shallow to be evaluated in terms of temperature dependence of electrical properties^41^, and acceptor defects exceeding 10^17^ cm^–3^ hinders forming the Schottky contacts required for the measurements including deep level transient spectroscopy^42^, which were commonly used for bulk-Ge^26,27^. In this study, by employing our SPC technique, the acceptor defects in poly-Ge thin films were explored, and are explained in this paper, in detail, by comparing their crystalline and electrical properties. Our proposed analysis method based on linear regression method clarified that the investigated poly-Ge thin films had two types of acceptor levels, one corresponding to intra-grain crystallinity and the other, to GBs.

## Experimental

Ge layers were deposited on SiO_2_ glass substrates using the Knudsen cell of a molecular beam deposition system (base pressure: 5 × 10^–7^ Pa), at a deposition rate of 1.0 nm min^–1^ and deposition time of 100 min. The Ge source, manufactured by Furuuchi Chemical Corporation, had a purity of 99.999%. To form poly-Ge layers with various crystallinities^35^, the substrate temperature during the deposition *T*_d_ was varied from 50 to 200 °C. We noted that *T*_d_ spontaneously rose from the ambient temperature to 50 °C even without the substrate being heated owing to the heat propagation from the Knudsen cell. The samples were then loaded into a conventional tube furnace in an N_2_ atmosphere and annealed at 450 °C for 5 h to induce SPC; this was followed by post annealing (PA) at 500 °C for 5 h in an Ar atmosphere.

The resulting samples were evaluated using electron backscatter diffraction (EBSD), Raman spectroscopy, and Hall effect measurements. The EBSD analyses were performed using a JEOL JSM-7001F (voltage: 25 kV; current: 15 mA) with a TSL OIM analysis attachment. The Raman spectra were measured using a JASCO NRS-5100 with a frequency-doubled Nd:YAG laser (wavelength: 532 nm; spot diameter: 20 μm; power: 0.5 mW), wherein the power was sufficiently low as to not affect the Ge crystallinity. The Hall effect measurements with the Van der Pauw method were performed using a Bio-Rad HL5500PC with a 0.32 T permanent magnet; here, the measurement temperature was varied from 115 to 400 K. Clear ohmic contacts were obtained simply by bringing the measurement probes into contact with the samples, without forming electrodes, which is owing to the strong fermi-level pinning^4^ and low resistance due to acceptor defects in poly-Ge^28,29^. The linear regression simulations were performed using the Python SciPy library.

## Results and discussion

**Figure 1:**
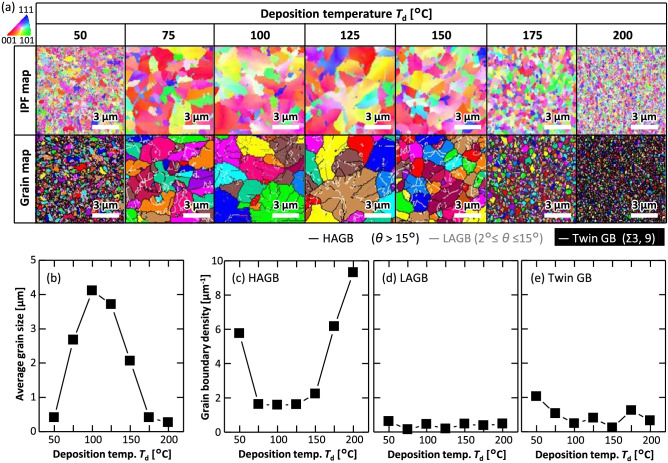
EBSD analysis of the Ge layers. (**a**) IPF and grain maps of the Ge layers as a matrix of *T*_d_. The colors in the IPF maps indicate crystal orientation, following the color key provided. (**b**) Average grain size as a function of *T*_d_. Grain boundary density as a function of *T*_d_ of (**c**) HAGBs, (**d**) LAGBs, and (**e**) twin GBs.

Figure 1a shows the inverse pole figure (IPF) and grain maps. The IPF maps indicate that the crystal orientation was generally random in all the samples. The grain size varied significantly with *T*_d_, as shown in the grain maps. This is because the modulation of the density of the Ge precursor changed the lateral growth rate in the SPC process^35^. For samples with large grain sizes (75 ≤ *T*_d_ ≤ 150 °C), GBs were noticeable within the grains; these were classified as high-angular GBs (HAGBs; mis-orientation angle *θ* > 15°), low-angular GBs (LAGBs; 2° ≤ *θ* ≤ 15°), and twin GBs (Σ3 and Σ9 coincidence site lattice). A comparison with the IPF maps suggests that these GBs were produced during the grain-growth process. The area-weighted average grain sizes are depicted in Fig. 1b, wherein the grains were defined as the area surrounded by random GBs. The grain size peaked at *T*_d_ = 100 °C, which was determined by the balance between the amorphous densification and crystal nucleation during the heat deposition. Figure 1c–e show that the HAGB was the most dominant among the GBs. The density of the HAGBs was highly dependent on *T*_d_ and correlated with the grain size (Fig. 1c). LAGBs were less abundant than the HAGBs, and no clear dependence on *T*_d_ was evident (Fig. 1d). The twin GBs tended to increase with a lower *T*_d_ (Fig. 1e). A low *T*_d_ would promote a slow growth velocity^35^, which might lead to the generation of twin GBs with low formation energies^43,44^.

**Figure 2:**
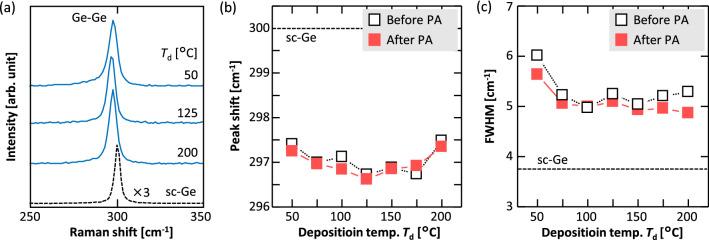
Raman spectroscopy study of the Ge layers. (**a**) Raman spectra of the samples before PA. (**b**) Raman shifts and (**c**) FWHMs of the Ge peaks of the samples before and after PA as functions of *T*_d_. The data for a bulk sc-Ge wafer are shown by the dotted lines.

Figure 2a shows sharp Raman peaks attributed to the polycrystalline Ge for various *T*_d_ values. The peak positions shifted to a lower wavenumber than that in the sc-Ge substrate (300 cm^–1^). This indicates that a tensile strain acted on the Ge layer^45^; this strain originated from the amorphous-to-crystalline phase transition and thermal expansion coefficient difference between Ge and the substrate^15^. Figure 2b shows that the Raman shift of the Ge peak first decreased and then increased with increasing *T*_d_. From a comparison of the grain sizes (Fig. 1), it appears that a larger grain size provided a larger shift corresponding to a larger tensile strain. This was possibly because the GBs mitigated the strain. PA at 500 °C tended to increase the amount of this shift. This tendency was more noticeable in the large-grained samples, possibly because the tensile strain caused by the thermal expansion difference was promoted by the high-temperature annealing. Figure 2c shows that the full width at half-maximum (FWHM) of the Ge peak was especially large for *T*_d_ = 50 °C and remained almost constant for *T*_d_ > 50 °C. The large FWHM at *T*_d_ = 50 °C was consistent with the asymmetry of the Raman spectrum (Fig. 2a), indicating a disorderly behavior in the atomic arrangement of the Ge crystal. These results suggest that the densification of the precursor by heat deposition enhanced the intra-grain crystallinity as well as the grain size. The low FWHM obtained even at *T*_d_ = 200 °C with a small grain size suggests that the FWHM mainly reflected the intra-grain crystallinity. Even though the grain size did not change after PA, the latter decreased the FWHM for all *T*_d_ values, which was possibly due to the improvement in the intra-grain crystallinity. Thus, *T*_d_ and PA significantly affected the crystallinity (i.e., GB density and/or inter-grain quality) of the Ge layers.

**Figure 3:**
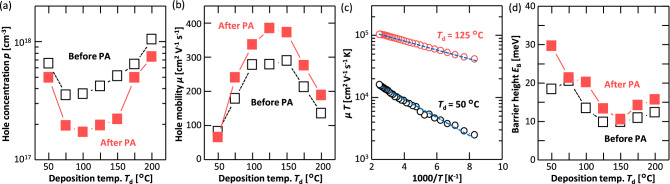
Electrical properties of the Ge layers as a function of *T*_d_ before and after PA. (**a**) *p* and (**b**) *μ*, which were averaged over four measurements for each sample. (**c**) Arrhenius plots of *μT* of the samples for *T*_d_ = 50 and 125 °C before PA; here, the dotted lines are the fitted lines used to derive *E*_B_. (**d**) *E*_B_ of GBs as a function of *T*_d_.

The Hall effect measurements revealed that the electrical conduction exhibited was entirely p-type owing to the acceptor defects in Ge^20–27^. Figure 3a shows that *p* considerably depended on *T*_d_, as the behavior of *p* was strongly related to the density of the HAGBs (Fig. 1c). Figure 3b shows that the hole mobility *μ* also depended on *T*_d_. *μ* increased with increasing grain size, which is generally true of polycrystalline semiconductor thin films^28,33,46–48^. From the measurement temperature *T* dependence of *μ*, we investigated the derivation of the energy barrier height of the GB ${E}_{\mathrm{B}}$. The most common model proposed by Seto assumes carrier transport only near grain boundaries^46^, which is not appropriate for use in the current Ge layers with μm order grain size. Therefore, we analyzed *E*_B_ using the model proposed by Evans and Nelson, which considers carrier transport within grains in addition to grain boundaries^47,48^. According to the model, *μ* limited by GB scattering can be determined using the following:

1 $$\begin{array}{c}\mu =\frac{Lq}{{k}_{\mathrm{B}}T}\frac{{v}_{r}}{1+\frac{{v}_{r}}{{v}_{d}}}\mathrm{exp}\left(-\frac{{E}_{\mathrm{B}}}{{k}_{\mathrm{B}}T}\right),\end{array}$$

where $L$ is the grain size; *q* is the elementary charge; ${v}_{r}$ is the recombination velocity; ${v}_{d}$ is the drift–diffusion velocity; and ${k}_{\mathrm{B}}$ is the Boltzmann constant. Figure 3c shows an Arrhenius plots of *μ*$T$. For both *T*_d_ = 50 °C and 125 °C, the dataset is a right–down straight line, and can be fitted with Eq. (1) in the entire region; this indicates that *μ* was dominantly determined by the GB scattering. ${E}_{\mathrm{B}}$ in Fig. 3d, calculated from the slope of these lines, was strongly influenced by *T*_d_. The grain size (Fig. 1b) and ${E}_{\mathrm{B}}$ (Fig. 3d) clearly explain the behavior of *μ* with respect to *T*_d_ (Fig. 3b). PA reduced *p* and increased *μ* for almost all *T*_d_ values, which was more pronounced for the samples with larger grain sizes (75 ≤ *T*_d_ ≤ 150 °C) (Fig. 3a,b). Considering that PA did not change ${E}_{\mathrm{B}}$ significantly (Fig. 3d), the decrease in *p* due to PA was responsible for the increase in *μ* due to the decease of the impurity scattering^15,36^.

**Figure 4:**
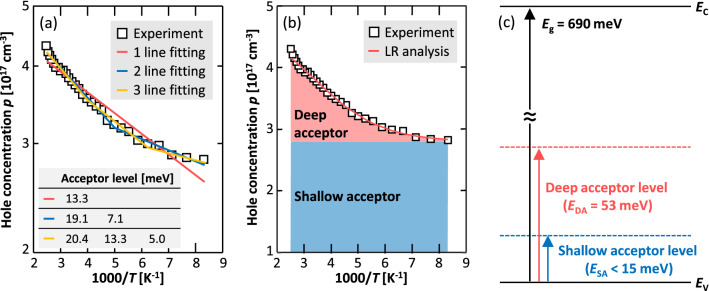
Characterization of the acceptor levels for the sample with *T*_d_ = 125 °C before PA. (**a,b**) Dependence of *p* on the measured temperature *T*, analyzed using (**a**) conventional linear fitting and (**b**) linear regression (LR) analysis. The acceptor levels determined by the linear fitting are shown in (**a**). (**c**) Acceptor levels shown in the bandgap structure of Ge, determined by the linear regression simulation.

The dependence of *p* on *T* was measured for all samples to determine the acceptor level in the poly-Ge layers. Figure 4a shows a typical result, wherein, *p* decreases with *T*, thus reflecting the inactivation of the acceptor^1,20^. In general, the acceptor level is estimated from a linear approximation of the slope in an Arrhenius plot of *p* and the acceptor concentration from the saturation value^49^. However, the dataset shown in Fig. 4a is represented by a curve that does not contain any linear or saturated regions; furthermore, this behavior was the same across all the samples. These results suggest that the poly-Ge layers contained multiple acceptor levels. Hofmann’s method^49^ and the free-carrier concentration spectroscopy method^50^ were used to estimate multiple acceptor levels. However, because the range of variation of *p* with *T* was small in the poly-Ge layers (implying shallow acceptor levels), the conventional linear fitting methods have the following problems: (i) It is unclear how many lines should be fitted (i.e., how many levels there are). (ii) Fitting using multiple lines involves subjectivity because the boundary of the *T* range to be fitted is unclear. (iii) The lines are not independent of each other owing to the close proximity of *p*, which complicates the correct derivation of the respective acceptor levels. The acceptor levels determined by the conventional linear fitting method were 5–20 meV (Fig. 4a). Considering that the energy in the *T* range is above 15 meV (equivalent to 115 K), estimating such small levels is difficult in principle and the values are unreliable. Therefore, it was difficult to derive a single fitting solution even by applying the conventional linear fitting methods.

Therefore, we proposed a simple and fast analysis method based on linear regression, which is commonly used in machine learning^51^. A function *p*(*T*), which originates from the fully ionized acceptor levels, can be expressed as follows:

2 $$\begin{array}{c}\begin{array}{c}p\left(T\right)=\sum\limits_{j=1}^{n}{N}_{j}f\left({{E}_{\mathrm{F}}-E}_{j},T\right)\end{array}\end{array}$$

3 $$\begin{array}{c}\begin{array}{c}f\left({E}_{\mathrm{F}}-E,T\right)=\frac{1}{1+\mathrm{exp}\left(-\frac{{E}_{\mathrm{F}}-E}{{k}_{\mathrm{B}}T}\right)}\end{array} ,\end{array}$$

where the degeneracy factor of the acceptor level was set to one, and the electron concentration was assumed to be sufficiently small compared to *p* that it can neglected. ${N}_{j}$ and ${E}_{j}$ are the density and energy levels of the *j*-th fully ionized acceptor level, respectively, *n* is the number of acceptor levels. The Fermi level ${E}_{\mathrm{F}}$ can be obtained numerically by solving the following integral equation using the Newton’s method:

4 $$\begin{array}{c}p\left(T\right)={\int }_{{E}_{V}^{\mathrm{bottom}}}^{{E}_{V}^{\mathrm{top}}}4\pi {\left(\frac{2m}{{h}^{2}}\right)}^\frac{3}{2}\sqrt{{E}_{V}^{\mathrm{top}}-E} \left[1-f\left({E}_{\mathrm{F}}-E,T\right)\right]dE ,\end{array}$$

where *m* is the effective mass of the hole; $h$ is Planck's constant; and ${E}_{V}^{\mathrm{top}}$ and ${E}_{V}^{\mathrm{bottom}}$ are the upper and lower edges of the valence band, respectively. Because the tensile strain of the Ge layers estimated from the Raman analyses in Fig. 2 was sufficiently small not to affect *m*^52^, we used the *m* value of strain-free Ge, which is determined by the following equation:

5 $$\begin{array}{c}m={\left({m}_{hh}^\frac{3}{2}+{m}_{lh}^\frac{3}{2}\right)}^\frac{2}{3},\end{array}$$

where the heavy hole mass ${m}_{hh}$ and light hole mass ${m}_{lh}$ were expressed to be 0.3*m*_0_ and 0.04*m*_0_*,* respectively, using the free electron mass *m*_0_^53^. Let the measured temperature points be $T$ = ${T}_{1}$, ${T}_{2}$, …, ${T}_{m}$, and *p* be collectively denoted as ${\varvec{Y}}$ = (*p*(${T}_{1}$) *p*(${T}_{2}$) … *p*(${T}_{m}$)); then, Eq. (2), is equivalent to the following equations:

6 $$\begin{array}{c}\begin{array}{c}Y={{\varvec{W}}}^{t}X . \end{array}\end{array}$$

7 $$\begin{array}{c}\begin{array}{c}W= \left(\begin{array}{c}{N}_{1}\\ \vdots \\ {N}_{n}\end{array}\right).\end{array}\end{array}$$

8 $$\begin{array}{c}\begin{array}{c}X= \left(\begin{array}{cccc}f\left({{E}_{\mathrm{F}}-E}_{1}, {T}_{1}\right)& f\left({{E}_{\mathrm{F}}-E}_{1}, {T}_{2}\right)& \cdots & f\left({{E}_{\mathrm{F}}-E}_{1}, {T}_{m}\right)\\ f\left({{E}_{\mathrm{F}}-E}_{2}, {T}_{1}\right)& f\left({{E}_{\mathrm{F}}-E}_{2}, {T}_{2}\right)& \cdots & f\left({{E}_{\mathrm{F}}-E}_{2}, {T}_{m}\right)\\ \vdots & \vdots & & \vdots \\ f\left({{E}_{\mathrm{F}}-E}_{n}, {T}_{1}\right)& f\left({{E}_{\mathrm{F}}-E}_{n}, {T}_{2}\right)& \dots & f\left({{E}_{\mathrm{F}}-E}_{n}, {T}_{m}\right)\end{array}\right)\end{array} .\end{array}$$

Equation (6) can be interpreted as a linear regression model, where ${\varvec{X}}$ is the explanatory variable and ${\varvec{W}}$ is the coefficient matrix. Given a set of levels $\left\{{E}_{j}\right\}$, the optimization problem of ${\varvec{W}}$ can be solved quickly^51^. Therefore, a brute-force search with a range of acceptor levels and energy intervals can be performed to obtain ${N}_{j}$ and ${E}_{j}$ with a processing time of a few seconds; that is, we solved Eq. (2) as the following optimization problem:

9 $$\begin{array}{c}\begin{array}{c}\underset{{E}_{1}, \dots , {E}_{n}}{\mathrm{argmin}}\left\{\underset{{N}_{1} , \dots ,{ N}_{n}}{\mathrm{min}}\frac{1}{2}\sum\limits_{i=1}^{m}{\left[p\left({T}_{i}\right)-\sum\limits_{j=1}^{n}{N}_{j}f\left({{E}_{\mathrm{F}}-E}_{j}, {T}_{i}\right)\right]}^{2}\right\}\end{array}. \end{array}$$

The above approach was adapted to the datasets shown in Fig. 4b, and the results were in a very good agreement with the linear regression analysis curve, including the two acceptor levels, one of which we defined as a deep acceptor level and the other a shallow acceptor level. The acceptor emitted by the deep acceptor level (*E*_DA_) was strongly dependent on *T*, whereas that emitted by the shallow acceptor level (*E*_SA_) was almost constant in this *T* range, as illustrated in Fig. 4b. This behavior indicates that the *T* range corresponded to its extrinsic region, as *E*_SA_ was less than the energy (15 meV), equivalent to the lower limit of *T* in this case (115 K). On the other hand, the optimum *E*_DA_ value was determined to be 53 meV, as shown in Fig. 4c. Thus, we propose a linear regression analysis method, which clarifies that the poly-Ge layers contain two types of acceptor levels, as illustrated in Fig. 4c.

**Figure 5:**
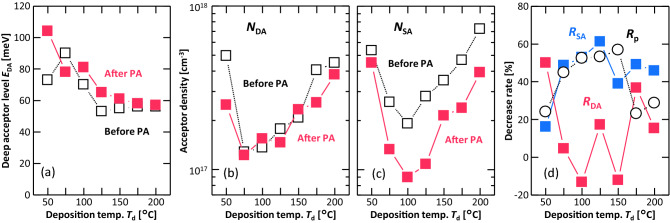
Analyses of the acceptors in the Ge layers before and after PA as a function of *T*_d_. (**a**) Deep acceptor level (*E*_DA_). Densities of (**b**) deep acceptor (*N*_DA_) and (**c**) shallow acceptor (*N*_SA_) layers. (**d**) Decrease rates of *N*_DA_, *N*_SA_, and *p* (*R*_DA_, *R*_SA_, and *R*_p_, respectively).

Using the proposed analysis method, *E*_DA_ was determined for all the samples. Figure 5a shows that *E*_DA_ was in the range of 53–103 meV and decreased with increasing *T*_d_. PA did not cause significant changes in *E*_DA_. These trends are similar to those of *E*_B_ (Fig. 3d). Figure 5b,c show the densities of the deep acceptor (*N*_DA_) and shallow acceptor (*N*_SA_) layers, which were derived from the analysis. Both *N*_DA_ and *N*_SA_ were lower for samples with larger grain sizes (75 ≤ *T*_d_ ≤ 150 °C), which was consistent with the behavior of *p* (Fig. 3a). On the other hand, the effect of PA on *N*_SA_ and *N*_DA_ was different with respect to *T*_d_; that is, PA reduced *N*_DA_ in the small-grained samples, while PA reduced *N*_SA_ in the large-grained samples. For a systematic understanding of the effect of PA, we defined the rate of density decrease *R* due to PA as follows:

10 $$\begin{array}{c}R=\frac{{D}_{1}-{D}_{2}}{{D}_{1}} ,\end{array}$$

where ${D}_{1}$ and ${D}_{2}$ are the densities (*N*_DA_, *N*_SA_, and *p*) before and after PA, respectively. Based on Eq. (10), *R* values of *N*_DA_, *N*_SA_, and *p,* defined as *R*_DA_, *R*_SA_, and *R*_*p*_, respectively, are shown in Fig. 5d. *R*_DA_ was larger in small-grained samples (*T*_d_ < 75 °C or *T*_d_ > 150 °C), whereas *R*_SA_ and *R*_*p*_ are larger in large-grained samples (75 ≤ *T*_d_ ≤ 150 °C). These results indicate that the decrease in *p* by PA was mainly owing to the decrease in *N*_SA_.

Based on the above results, we phenomenologically discuss the origin of the acceptor defects in polycrystalline Ge. According to the previous studies, the physical origin of acceptors in Ge was speculated to be vacancies and dangling bonds^19–27^. The correlation between the HAGBs (Fig. 1c) and *p* (Fig. 3a) suggests that the dangling bonds located at HAGBs are one of the origins of the acceptors. However, a larger grain size (Fig. 1b) led to a larger *R*_*p*_ (Fig. 5d), implying that the intra-grain crystallinity also had acceptors, which were reduced by PA. This is consistent with the observation that crystallinity was enhanced by PA, as determined from the Raman spectra (Fig. 2c). The observation that a larger grain size led to a smaller *R*_DA_ and a larger *R*_SA_ suggests that the deep acceptor level could be attributed to the dangling bond located at HAGBs, while the shallow acceptor level, to the vacancies in grains (Fig. 5d). The similarity between Figs. 3d and 5d is reasonable if the deep acceptor level was caused by the dangling bond located at HAGBs: a larger *E*_DA_ (Fig. 5a) would reduce *p* near the HAGBs and yield a larger *E*_B_ (Fig. 3d) because *E*_B_ was inversely proportional to *p*^46–48^. Thus, the acceptor defects in the poly-Ge layers consisted of deep acceptors (53–103 meV) originating from the dangling bond located at HAGBs, and shallow acceptors (< 15 meV) originating from the vacancies in grains. These results are generally consistent with theoretical calculations and experimental results for bulk Ge, wherein it has been observed that the dangling bond located at HAGBs form relatively deep acceptor levels (approximately 60 meV)^19^, while vacancies form shallow acceptor levels (14–25 meV)^22^.

## Conclusions

We experimentally estimated the origin of the acceptor defects in the poly-Ge layers. The grain size and electrical properties of the poly-Ge layer were extensively modulated by controlling *T*_d_ in the SPC. Moreover, PA, performed at 500 °C also had a significant effect on the electrical properties. We proposed a linear regression analysis method to derive the acceptor levels and densities from the temperature dependence of *p*, which revealed the existence of two types of acceptor level. The effects of grain size and PA on *p* were systematically investigated, which suggest that the deep acceptor levels (53–103 meV) could be attributed to the dangling bonds located at HAGBs, while the shallow acceptor levels (< 15 meV), to the vacancies in grains. These findings will contribute to the understanding as well as methods to mitigate acceptor defects, which hinder the application of poly-Ge layers in semiconductors.

## Data Availability

The datasets used and/or analyzed during the current study available from the corresponding author on reasonable request.
